# Screening and Evaluation of Potential Efflux Pump Inhibitors with a Seaweed Compound Diphenylmethane-Scaffold against Drug-Resistant *Escherichia coli*

**DOI:** 10.3390/antibiotics13070628

**Published:** 2024-07-05

**Authors:** Wen-Jung Lu, Yu-Wei Lian, Chun-Ju Chang, Hsuan-Ju Lin, Chian-Yun Huang, Pang-Hung Hsu, Hong-Ting Lin

**Affiliations:** 1 Department of Food Science, National Taiwan Ocean University, Keelung 202, Taiwan; wen-jung.lu@nottingham.ac.uk (W.-J.L.); 11032024@mail.ntou.edu.tw (Y.-W.L.); chunju@mail.ntou.edu.tw (C.-J.C.); 20532001@mail.ntou.edu.tw (H.-J.L.); 11132012@mail.ntou.edu.tw (C.-Y.H.); 2 School of Life Sciences, University of Nottingham, Queen’s Medical Centre, Nottingham NG7 2UH, UK; 3 Center of Excellence for the Oceans, National Taiwan Ocean University, Keelung 202, Taiwan; 4 Department of Bioscience and Biotechnology, National Taiwan Ocean University, Keelung 202, Taiwan

**Keywords:** efflux pump inhibitors, multidrug resistance, drug transporters, drug efflux, molecular docking

## Abstract

Drug-resistant efflux pumps play a crucial role in bacterial antibiotic resistance. In this study, potential efflux pump inhibitors (EPIs) with a diphenylmethane scaffold were screened and evaluated against drug-resistant *Escherichia coli*. Twenty-four compounds were docked against the drug-binding site of *E. coli* multidrug transporter AcrB, and 2,2-diphenylethanol (DPE), di-*p*-tolyl-methanol (DPT), and 4-(benzylphenyl) acetonitrile (BPA) were screened for their highest binding free energy. The modulation assay was further used for EPI evaluation, revealing that DPE, DPT, and BPA could reduce the drug IC_50_ value in *E. coli* strains overexpressing AcrB, indicating their modulation activity. Only DPE and BPA enhanced intracellular dye accumulation and inhibited the efflux of ethidium bromide and erythromycin. In addition, DPE and BPA showed an elevated post-antibiotic effect on drug-resistant *E. coli*, and they did not damage the permeability of the bacterial outer membrane. The cell toxicity test showed that DPE and BPA had limited human-cell toxicity. Therefore, DPE and BPA demonstrate efflux pump inhibitory activity, and they should be further explored as potential enhancers to improve the effectiveness of existing antibiotics against drug-resistant *E. coli*.

## 1. Introduction

Drug resistance, also known as antimicrobial resistance, is a phenomenon in which microbes, such as bacteria, viruses, and parasites, evolve and adapt to drugs that were previously found to be effective in killing such microbes. This phenomenon causes these drugs to become less effective over time, which is a cause for global concern because of its implications for public health. The World Health Organization has indicated that drug resistance is an important issue for human health because it threatens the effectiveness of treatments for an increasing range of infections [1]. Bacterial AMR was responsible for 1.27 million global deaths in 2019, and it contributed to 4.95 million deaths [2]. However, the development of new antibiotics has been slowing down recently because of multiple factors. The complex nature of antibiotic development, combined with financial and regulatory challenges, has led to a decline in the number of pharmaceutical companies involved in antibiotic research and development [3].

Bacterial drug efflux pumps are structures that majorly contribute to the mechanisms of antibiotic resistance. These protein transporters actively transport certain drugs out of the bacterial cell, thereby decreasing the intracellular concentration of the drug and rendering it ineffective [4]. NorA, which is found in *Staphylococcus aureus*, is a prominent example of an efflux pump [5,6]. NorA confers resistance to various antibiotics, such as fluoroquinolones, biocides, and dyes. Another notable example is the AcrAB-TolC tripartite efflux pump system, which is found in many gram-negative bacteria, such as *Escherichia coli* and *Salmonella* [4,7]. This AcrAB-TolC system removes a broad range of antibiotics, including tetracycline, chloramphenicol, and fluoroquinolones, and it is considered a primary mechanism of multidrug resistance in these bacteria. Efflux pump inhibitors (EPIs) are a distinct class of therapeutics designed specifically to obstruct the function of efflux pumps localized in cytoplasmic membranes [8]. By blocking these pumps, EPIs can prevent the bacteria from expelling the antibiotic molecule, allowing the drug to remain in the cell and effectively kill the bacteria [9,10]. At present, chemically synthesized EPIs have been extensively studied for their potential application in combating drug resistance. Phenyl-arginine-β-naphthylamide (PAβN, MC-207,110), which was identified in 2001 as a broad-spectrum EPI, has bacteriostatic and cytotoxic properties. PAβN is known to inhibit the AcrB efflux pump in *E. coli* [11]. D13-9001 is an inhibitor of the MexAB-OprM efflux pump in *Pseudomonas aeruginosa* that has low cytotoxicity, and it consists of a pyridine and pyrimidine core structure. Studies have shown that D13-9001 binds to the deep binding pocket regions of MexB and AcrB, inhibiting the efflux of drugs by MexAB-OprM and AcrAB-TolC [12]. In terms of natural compounds with EPI activity, Dias et al. [13] indicated that eugenol can interfere with the drug transport of the NorA efflux pump in *S. aureus*. Berberine can remarkably increase the intracellular concentration of ciprofloxacin and restore the sensitivity of the bacterial strain to antibiotics by inhibiting the MdfA efflux pump in *E. coli* [14]. However, although these compounds have demonstrated promising results in laboratory settings, none have gained approval for clinical use [15].

Seaweeds consist of a variety of biologically active compound activities [16]. Brown and red seaweed alcohol extracts serve as potential EPIs for drug-resistant *E. coli* [17]. Diphenylmethane (DPM), a natural compound identified in red seaweed *Gracilaria* sp., can interfere with efflux pump AcrB, increase intracellular antibiotic accumulation, and reinforce their effectiveness against drug-resistant *E. coli* [18]. In this study, we aimed to exploit molecular docking (Autodock vina) to screen chemical compounds with a DPM scaffold for better binding energy with efflux pump AcrB. We also aimed to further adapt experimental approaches to evaluate their efflux pump activity against drug-resistant *E. coli*. Supplementary Materials Figure S1 displays the experimental design schematic diagram for the investigation of diphenylmethane-scaffold seaweed compounds as potential efflux pump inhibitors against drug-resistant *Escherichia coli*.

## 2. Results and Discussion

### 2.1. Molecular Docking

Previous structural studies have identified the drug-binding pockets, drug entrance, and efflux pathways of the AcrB transporter, thereby providing valuable insights [19,20]. The performed molecular docking using Autodock Vina revealed varying levels of binding affinity of the tested compounds to the drug-binding pockets of the *E. coli* RND drug transporter AcrB. The docking region was selected inside the distal binding pocket and proximal binding pocket of the tight state of the AcrB monomer, and the interaction residues for each compound were complex and varied.

**Figure 1:**
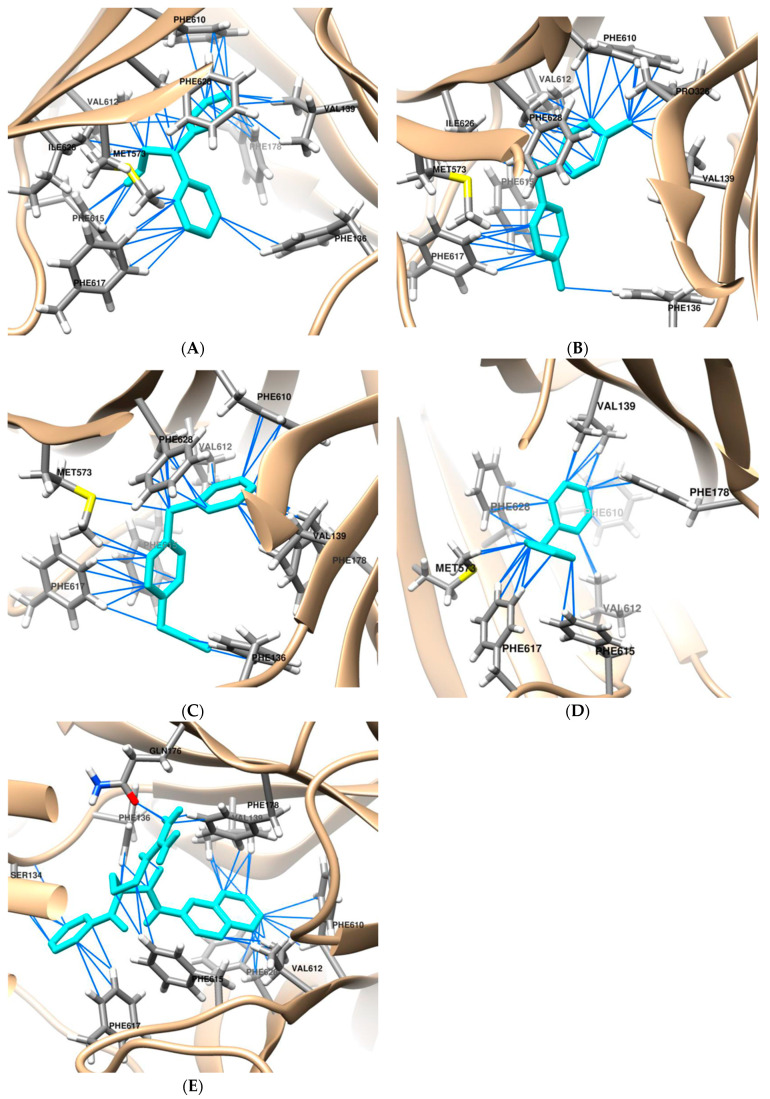
The binding model of DPM derivatives into the AcrB efflux pump (**A**) DPE, (**B**) DPT, (**C**) BPA, (**D**) DPM, and (**E**) PAβN. The DPM derivatives and PAβN are colored in cyan.

Among the 24 compounds with a DPM scaffold, the compounds DPE, DPT, and BPA were screened out for their higher binding affinities, with a binding free energy of −8.2, −8.2, and −8.5 kcal/mol, respectively (Table 1). The AcrB inhibitor PAβN showed substantial binding affinities with a binding free energy of −8.8 kcal/mol, and DPM exhibited a binding free energy of −8.1 kcal/mol. In addition, DPE, DPT, and BPA showed similar levels of pseudobond (32–43) and van der Waals force (8–10) with AcrB, and comparable binding affinities. The compounds were stabilized through hydrogen bonding and hydrophobic interactions with phenylalanine residues. Figure 1 displays the docked poses and binding of PAβN, DPM, DPE, DPT, and BPA. When comparing the interaction residues of the compounds (Table 2), many common were present among the compounds, such as Val139, Phe610, Val612, Phe615, and Phe617. The presence of these common residues indicates that they may play a crucial role in the binding of compounds to AcrB. Moreover, the distinct residues found for each compound could contribute to their unique binding affinities. For example, in the docking model, PAβN interacted with Ser134 and Gln176, which were absent in other compounds.

### 2.2. IC_50_ Assay for Antibiotics and DPE, and DPT and BPA against Drug-Resistant E. coli

The gram-negative pathogen *E. coli* is a challenging bacterial species in clinical therapies. Moreover, only a limited number of EPIs for gram-negative bacteria have been reported to date [21]. The strain *E. coli* Kam3 overexpressing the transporter AcrB (Kam3-AcrB) is used in this experiment. The antibiotics used are erythromycin, ciprofloxacin, clarithromycin, and tetracycline, which have been reported as the transport substrates of the efflux pump AcrB [22]. The results indicated that DPM, DPE, DPT, and BPA did not exhibit any remarkable bactericidal activity against the tested strains, and the IC_50_ value of these compounds represents the maximum soluble concentration of each chemical. The well-known inhibitor PAβN was used as the control group. The IC_50_ values of erythromycin, ciprofloxacin, clarithromycin, and tetracycline were determined to be 125, 0.015, 87.5, and 0.39 µg/mL, respectively. The MIC values of erythromycin, ciprofloxacin, clarithromycin, and tetracycline were reduced by two to fourfold with the combined use of DPE, DPT, and BPA, respectively (Table 3). Our IC_50_ and modulation results indicated that DPE, DTP, and BPA could modulate the activities of erythromycin, ciprofloxacin, clarithromycin, and tetracycline against Kam3-AcrB. Tambat et al. [23] indicated that an indole of microbial origin, namely, ethyl 4-bromopyrrole-2-carboxylate (RP1), could synergistically reduce the minimum inhibitory concentration of tetracycline, levofloxacin, and chloramphenicol against AcrAB overexpressing the strain *E. coli* AG100_tet_ by from 2-fold to 64-fold. Another potential EPI 4-(2-(piperazin-1-yl)ethoxy)-2-(4-propoxyphenyl) quinolone (PQQ4R) substantially enhanced the antibacterial effectiveness of tetracycline and ofloxacin in the AcrAB-overexpressing strain AG100_tet_ [24], although PQQ4R showed greater cytotoxicity than PAβN.

### 2.3. Pump Efflux Efficiency Reduced by DPE and BPA

Fluorescent assays have been frequently used to provide insights into the efflux of substrates by the detected cells expressing drug transporters. Accumulation and efflux assays were applied to study the efflux efficiency by monitoring the changes in fluorescence and erythromycin, and the relative fluorescence factor (RFF) was calculated for analysis. In this study, ethidium bromide (EB) and erythromycin were used to monitor the accumulation/efflux of the substrate in *E. coli* Kam3-AcrB, and the positive control group was established using the RND pump modulator PAβN [25]. Accumulation experiments (Figure S2) demonstrated that the presence of DPE and BPA resulted in a dose-dependent increase (higher RFF) in EB accumulation within *E. coli* Kam3-AcrB, whereas DPT showed a similar RFF as compared with the control (no addition of EPIs), providing indirect evidence that DPE and BPA could interfere with the efflux of EB by Kam3-AcrB (Table 4). Machado et al. [24] observed that PQQ4R, chlorpromazine, and PAβN displayed RFF values of 5.2, 7.9, and 1.3, respectively, against the AcrAB-overexpressing strain AG100_tet_, indicating that PQQ4R has higher levels of EB accumulation than PAβN.

**Figure 2:**
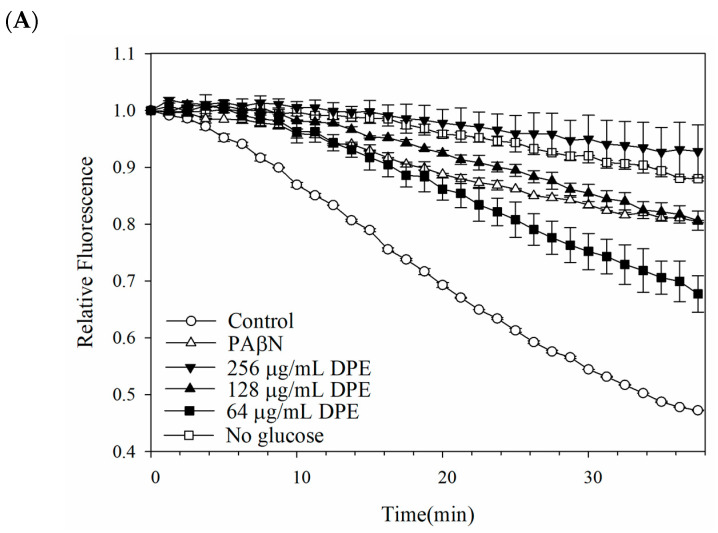

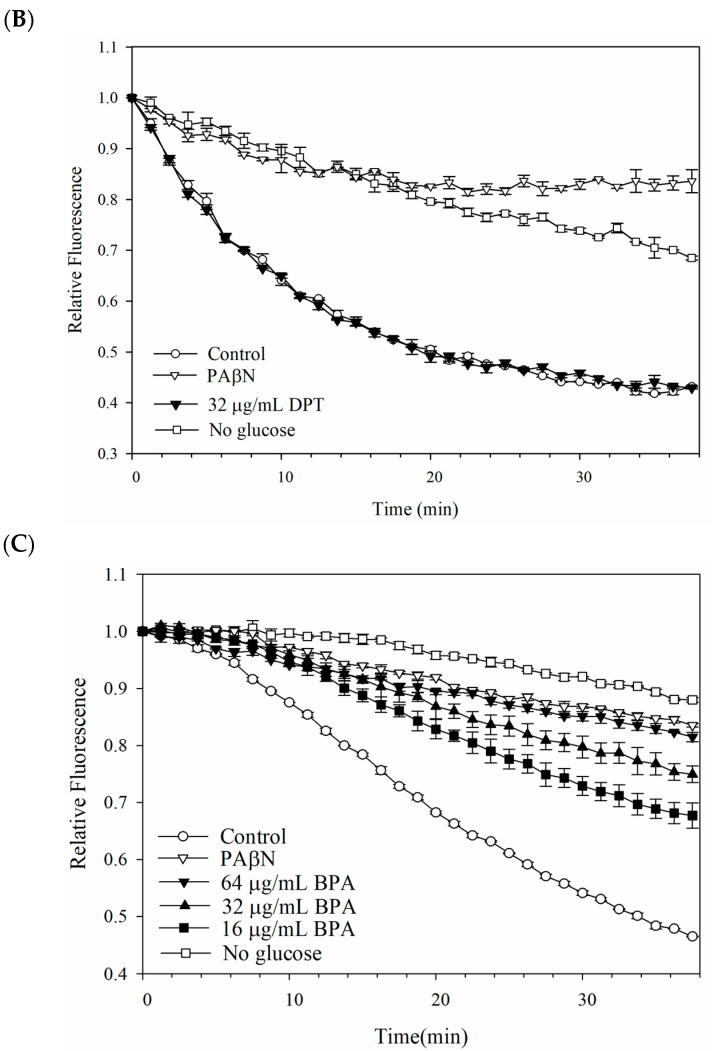
EB efflux inhibition assay of (**A**) DPE, (**B**) DPT, and (**C**) BPA in *E. coli* Kam3-AcrB.

The potential interference of a pump efflux inhibitor against *E. coli* Kam3-AcrB was further investigated by monitoring the EB efflux in Kam3-AcrB in the presence of DPE, DPT, and BPA. Figure 2 illustrates the gradual decrease in EB fluorescence by Kam3-AcrB (control) from time = 0 to reach a relative fluorescence of 0.46 in 38 min, indicating a steady efflux of EB from the *E. coli* cells. Additionally, the presence of the pump inhibitor PAβN resulted in a reduction in EB efflux, with a final relative fluorescence of 0.80 ± 0.01 at 38 min, indicating that PAβN could interfere with the efflux pump. The addition of DPE and BPA at IC_50_, 1/2 IC_50_, and 1/4 IC_50_ concentrations can slow the decrease of EB fluorescence in a dose-dependent manner. This effect is likely attributed to the reduced efflux of EB from the cell, which might be attributed to the interference caused by DPE and BPA with the AcrB efflux pump (Figure 2A,C). By contrast, the inclusion of DPT displayed an efflux level similar to Kam3-AcrB (control), indicating that the synergistic effect of DPT with antibiotics might not be caused by the interference with the efflux pump, which was consistent with the results of dye accumulation.

**Figure 3:**
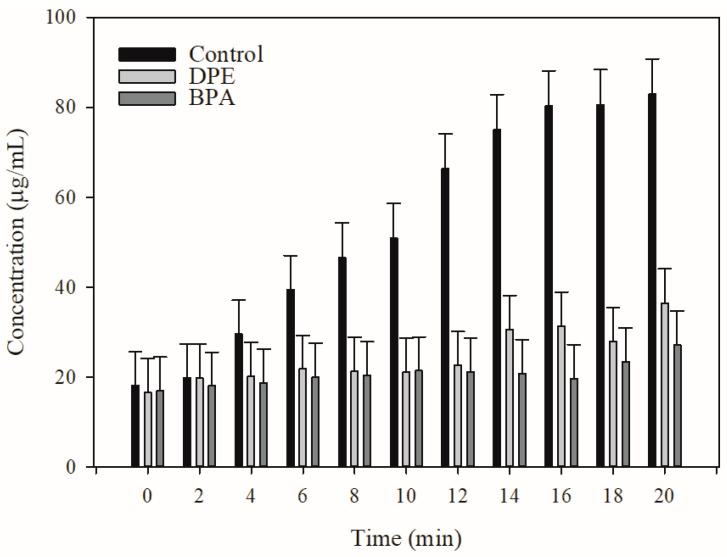
The use of MALDI-TOF MS allowed for the identification of the erythromycin efflux activity in *E. coli* Kam3-AcrB in the presence of DPM derivatives. The intensity of the main peak at m/z 738.35 of erythromycin was plotted, and the detection period lasted for 20 min. The results are presented as the mean± standard deviation (SD) (*n* = 3).

Based on the dye efflux data, DPE and BPA could disrupt dye efflux in *E. coli* Kam3-AcrB. However, these findings do not conclusively demonstrate that EPE and BPA interfere with the antibiotic efflux in *E. coli*. Thus, MALDI-TOF MS was utilized to monitor the efflux of erythromycin in the extracellular space with the addition of DPE and BPA. Figure 3 shows that the extracellular erythromycin concentration of Kam3-AcrB cells experienced a remarkable increase from 18.14 μg/mL (t = 0 min) to 82.89 μg/mL (t = 20 min) in the absence of DPE and BPA, indicating the continuous efflux of erythromycin by the Kam3-AcrB cells. Conversely, in the presence of DPE and BPA, the extracellular erythromycin concentration increased from 16.62 and 16.95 μg/mL (t = 0 min) to 36.45 and 27.08 μg/mL (t = 20 min), respectively, indicating a decrease in the efflux of erythromycin. Our data indicated that DPE and BPA could interfere with EB accumulation as well as erythromycin and EB efflux, thereby indicating their potential application as EPIs for drug-resistant *E. coli*.

### 2.4. Outer Membrane Permeability of Kam3-AcrB

**Figure 4:**
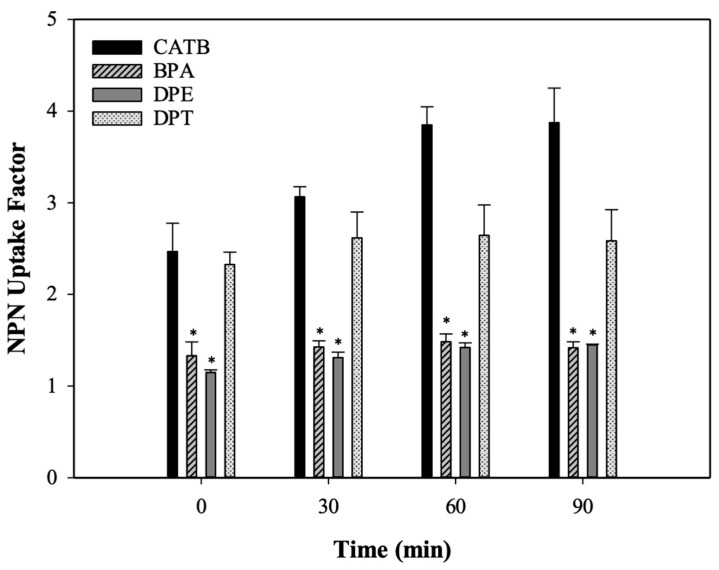
The uptake of NPN by Kam3-AcrB in the presence of DPM derivatives. CTAB, Cetyltrimethylammonium bromide; DPE (256 µg/mL); DPT (32 µg/mL); BPA (64 µg/mL). Data are expressed as mean ± SD (*n* = 3). The asterisk (*) indicates statistical significance from the CATB group.

Maintaining the integrity of the plasma membrane is of great importance to ensure the proper functioning and survival of cells. An increase in membrane permeability could disturb the proton motive force and destabilize the intracellular pH equilibrium. The NPN uptake test was utilized to investigate the effect of DPE, DPT, and BPA on the outer membrane permeabilization in Kam3-AcrB. Generally, the outer membrane serves as a barrier against the entry of NPN, which is a neutral hydrophobic fluorescent probe. However, the fluorescence intensity of NPN increases as it crosses the outer membrane. CTAB, a cationic surfactant, was utilized as a positive control to disrupt the cell membrane. Figure 4 demonstrates that NPN uptake factors increase in the presence of CTAB and DPT, suggesting the permeabilization of the outer membrane of Kam3-AcrB. By contrast, the NPN uptake factors of DPE and BPA were closer to 1 after 90 min incubation, indicating that the membrane permeability was maintained compared with the control (no addition). Tambat et al. [23] indicated that the addition of RP1 in the NPN assay exhibited a low level of fluorescence compared with the treatment of polymyxin B, a well-known membrane permeabilizer. Our results indicate that the potentiating effect of DPE and BPA for antibiotics was not due to membrane permeabilization.

### 2.5. Post-Antibiotic Effect of DPE and BPA Coupled with Erythromycin on E. coli Kam3

The post-antibiotic effect (PAE) refers to the persistent inhibition of bacterial proliferation after exposure to an antimicrobial agent [26], and this effect can be assessed by determining the duration of no growth of the target organism once the antibiotic is removed. As shown in Table 5, the PAE values of erythromycin at 1/2 × IC_50_, 2 × IC_50_, and 6 × IC_50_ for Kam3-AcrB were 0.61, 1.00, and 1.10, respectively. When DPE was combined with 1/2 × IC_50_, 2 × IC_50_, or 6× IC_50_ erythromycin, the resulting PAE values were 1.09, 1.13, and 1.44, respectively. The combination use of BPA under the same erythromycin condition exhibited PAE values of 1.08, 1.21, and 1.9. These data indicate that the addition of DPE and BPA slightly affects the PAEs of erythromycin. In a previous study, the combination of 32 µg/mL 2-(2-aminophenyl) indole (RP2) with ciprofloxacin against *S. aureus* SA-1199B (NorA overexpressing) could markedly increase the PAE value from 1.43 h to 3.33 h, indicating that the potential inhibitor IITR08367 (50 μM) could increase the PAE of fosfomycin against *E. coli*-overexpressing efflux pump AbaF from *Acinetobacter baumannii* by 30 min [27]. To date, few studies have indicated that EPIs may prolong the PAE of antibiotics against gram-negative bacteria.

### 2.6. Cytotoxicity Test of DPE and BPA

**Figure 5:**
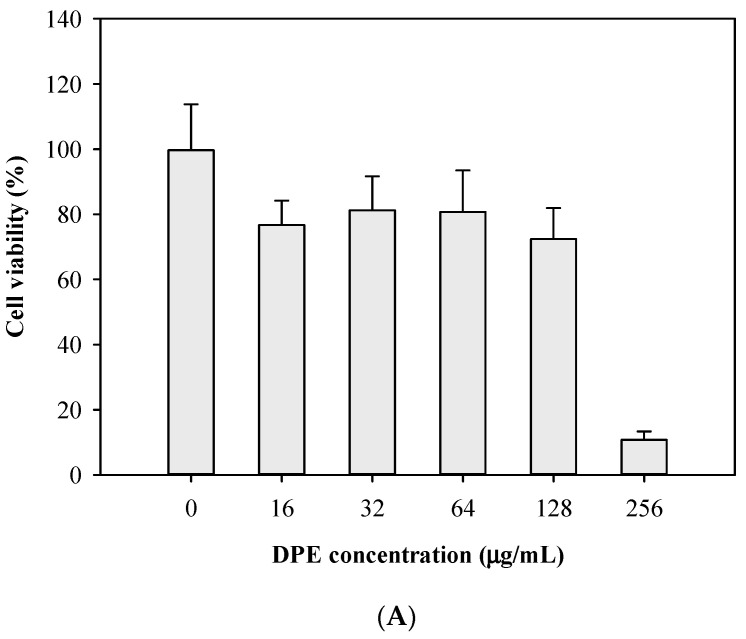

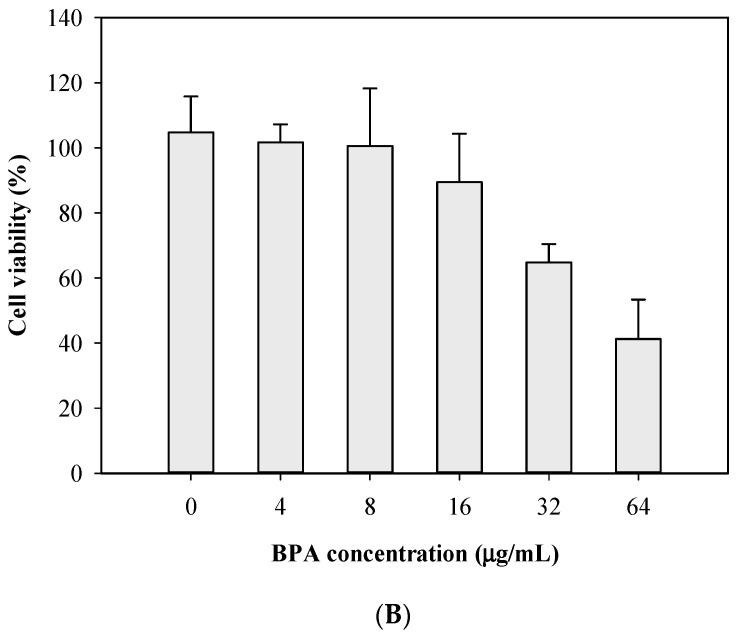
Cytotoxicity test of (**A**) DPE and (**B**) BPA at different concentrations with HepG2 cell. Data are expressed as mean ± SD (*n* = 3).

The potential cytotoxicity of the putative EPIs, namely, DPE and BPA, on human hepatocytes was assessed by observing the cell viability of HepG2 cells when exposed to different concentrations of DPE and BPA. HepG2 cells have both phase I and II drug-metabolizing enzymes [28]; as a result, HepG2 cells were routinely used in the evaluation of in vitro cytotoxic and genotoxic potential of chemicals and natural products [29]. As shown in Figure 5A, the cell viability was >80% at DPE concentrations of 16, 32, and 64 µg/mL, and the viability dropped to 72.4% and 10.8% when the DPE concentration was raised to 128 and 256 µg/mL, respectively. The cell viability for BPA at 4, 8, 16, 32, and 64 µg/mL was 101.7%, 100.6%, 89.4%, 64.8%, and 41.2% compared with the control group, and the cell viability gradually decreased as the BPA concentration increased to 32 μg/mL (Figure 5B). EPI is suggested to have ideal pharmacological features, such as non-toxicity, and high therapeutic and safety indices. A well-known ionophore EPI, carbonyl cyanide-m-chlorophenylhydrazone, can disrupt the proton motive force and may be toxic to mammalian cells. This has limited its use to the laboratory setting. Chandal et al. [30] reported a decrease in cell viability of human embryonic kidney HEK 293T cells when exposed to 250 and 500 μg/mL of the indole derivative, SMJ-5, accounting for 79.56% and 48.35% reductions, respectively. Our cytotoxicity data showed that the IC_50_ values of DPE and BPA against human HepG2 cells were determined to be 256 and 64 μg/mL (1.3 and 0.3 mM), respectively. In addition, our modulation assays demonstrated that DPE and BPA could enhance the effectiveness of antibiotics, such as erythromycin, ciprofloxacin, clarithromycin, and tetracycline, by two to fourfold against *E. coli* Kam3-AcrB at sub-IC_50_ doses (128 and 32 μg/mL) or lower. Therefore, DPE and BPA may revive antibiotic effectiveness at their sub-inhibitory concentration by potentially interfering with the drug efflux of *E. coli* Kam3-AcrB.

## 3. Materials and Methods

### 3.1. Bacterial Strains, Constructs, Media and Chemicals

*E. coli* Kam3 (DE3) lacking the *acrB* gene was utilized in drug susceptibility, modulation, and drug accumulation tests [31]. *E. coli* Kam3 (DE3) carrying the pSYC plasmid encoding acrB gene was utilized for drug susceptibility, checkerboard, drug accumulation, efflux inhibition, and time–kill assays. The bacteria were cultured in Mueller–Hinton broth (MH broth) and Luria–Bertani broth (LB) for growth and broth microdilution experiments. Ciprofloxacin, erythromycin, clarithromycin, tetracycline, diphenylmethane (DPM), 2,2-diphenylethanol (DPE), Di-*p*-tolyl-methanol (DPT), and 4-(benzylphenyl) acetonitrile (BPA) were purchased from Sigma-Aldrich (St. Louis, Missouri, USA). DPM, DPE, DPT, and BPA stock was prepared by dissolving in 95% ethanol with the concentration of 50, 25.6, 3.2, and 6.4 mg/mL, respectively, and they were diluted in PBS buffer and media for the experiments in this study.

### 3.2. Molecular Docking

Docking was carried out using Autodock Vina program 1.17 [32,33] with default parameters, and structure visualization was accomplished by using UCFS Chimera version 1.16 [34]. The crystal structure of the AcrB transporter (PDB database 4DX5, 1.9 Å) [35] was obtained from the Protein Data Bank [36] and used for the docking studies. The structure of 24 screening compounds (4-benzyl-benzaldehyde, 4-benzylaniline, 4,4′-Diaminodiphenylmethane, 4-benzylphenol, Bis(4-hydroxyphenyl)methane, benzophenone, benzhydrylamine, diphenylmethanol, (4-benzylphenyl)acetonitrile, di-*p*-tolyl-methanol, 2,2-diphenylethanol, 4-benzylbenzoic acid, 2-benzylphenol, 4,4′-methylenebis(N,N-dimethylaniline), 4-aminobenzophenone, 4,4′-diaminobenzophenone, 4-hydroxylbenzophenone, 4-(amino-*p*-tolyl-methyl)-phenol, 4-methoxybenzophenone, DL-4-methoxybenzhydrol, 4-hydroxy-4′-methoxybenzophenone methoxybenzophenone, diphenylacetic acid, 4-benzylphenyl carbamate, Michler’ ketone) were downloaded from PubChem (https://pubchem.ncbi.nlm.nih.gov) (accessed date 1 December 2021) and converted into a pdb file using Chimera 1.16. The AcrB was known to have three different conformation states during the substrate transport process, the docking region was chosen inside the DBP (distal binding pocket, DBP) and PBP (proximal binding pocket) of the tight state of the AcrB monomer with the search space of size 30 Å × 30 Å × 30 Å.

### 3.3. MIC and IC_50_ Determination

The MIC and IC_50_ experiments were conducted following the same procedure as described earlier but with some modifications [37,38]. To determine the minimum inhibitory concentration (MIC) and half-maximal inhibitory concentration (IC50) of DPM, DPE, DPT, and BPA against both drug-sensitive and drug-resistant *E. coli* strains, microdilution methods (MH broth) were employed with an inoculum of 10^5^ CFU/mL cell density. For the IC_50_ test, a twofold serial dilution series of each drug was prepared with MH broth in a flat-bottomed 96-well plate. The cell growth was then examined after a 12 h incubation at 37 °C using spectroscopy to determine the IC_50_. The same procedure was applied in the MIC test with a round-bottomed 96-well plate and the MIC was measured according to the CLSI guideline. The highest concentrations of DPM, DPE, DPT, and BPA were 500, 256, 32, and 64 μg/mL, respectively.

### 3.4. Modulation Assays

The potentiating efficacy of DPM derivatives with antibiotics was identified using modulation assay [18]. Briefly, antibiotics and tested compounds were diluted in 96 microplates with a series of twofold dilutions from MIC in two dimensions and mixed with 10^5^ CFU/mL bacteria culture following incubation at 37 °C for 12 h. The modulation factors for each antibiotic along with their EPI concentrations were recorded.

### 3.5. Drug Accumulation Assay

The ethidium bromide (EB) accumulation test was carried out based on previous investigations [39,40], with some modifications. *E. coli* cells were grown to mid-log phase in MH broth, then harvested through centrifugation (5000× *g*, 5 min and 4 °C). These cells were twice resuspended in phosphate-buffered saline (PBS) (10 mM Na_2_HPO_4_, 1.8 mM KH_2_PO_4_, 137 mM NaCl, 2.7 mM KCl, 1 mM CaCl_2_, and 0.5 mM MgCl_2_ at pH 7.4) and diluted in PBS to reach a final OD600 of 0.6. The cell suspension was placed in a 96-well plate with filter-sterilized glucose (25 mM) at room temperature for 3 min. Following the addition of EB (25 μM), the fluorescence was continuously monitored for 38 min. The excitation and emission wavelengths for EB were set at 520 nm and 600 nm. PAβN (final concentration of 20 μg/mL), DPM, DPE, DPT, and BPA were exposed to the bacterial suspension individually before fluorescence measurement. Relative Fluorescence Factor (RFF) = (RF_treated_-RF_untreated_)/RF_untreated_.

### 3.6. Efflux Inhibition Assay

The dye efflux assay was accomplished as described in a previously published study with the following modifications [41]. The *E. coli* Kam-AcrB cells were harvested by centrifugation (5000× *g*, 5 min, and 4 °C) after being grown to mid-log phase in MH broth and resuspended in PBS buffer twice. The cells were then diluted in PBS to a final OD600 of 0.6. Following an 8 h incubation at RT, the *E. coli* cells were exposed to EB (3 μM) for 30 min. Subsequently, the cells were resuspended twice in PBS buffer and incubated in 96-well plates with glucose (25 mM), PAβN (20 μg/mL), and varying concentrations of DPE, DPT, and BPA at RT, with fluorescence being monitored for 38 min.

### 3.7. Monitoring Drug Efflux Using MALDI-TOF Mass Spectrometry

Drug efflux was investigated using MALDI-TOF mass spectrometry (Bruker Daltonik GmbH, Bremen, Germany), employing a modified version of the established method [41]. Erythromycin was dissolved in 10 mM ammonium bicarbonate buffer at varying concentrations and mixed with matrix 2,5-dihydroxybenzoic acid for MALDI-TOF MS analysis to establish a standard curve. The *E. coli* Kam3-AcrB cells were cultured at 37 °C until reaching an OD600 of 0.6 to 0.8 in Mueller–Hinton broth, and then collected by centrifugation (6000× *g* for 5 min at 4 °C). After two washes, the cells were resuspended in ammonium bicarbonate buffer (pH = 7.5) to a final OD600 of 0.6. To evaluate the erythromycin efflux efficiency mediated by transporter AcrB using MALDI-TOF, the *E. coli* Kam3-AcrB cells were treated with erythromycin, DPE, BPA, and filter-sterilized glucose. Samples were collected at various time points over a 20 min duration. After collection, each sample from the respective time point underwent centrifugation at 6000× *g* for 1 min to obtain the supernatant for MS analysis. The samples were mixed with matrix 2,5-dihydroxybenzoic acid and analyzed by using MALDI-TOF MS. The data acquisition process was automated, employing the random walk mode, with 15 shots taken at each step, resulting in a total of 3000 shots per sample. The acquired mass spectra were analyzed by FlexAnalysis (version 3.0, Bruker Daltonics, Billerica, MA, USA), and the ion abundance of erythromycin in each spot was determined by integrating the signal.

### 3.8. Post-Antibiotic Effect Assay

This experiment was performed as previously described [42]. *E. coli* Kam3-AcrB was cultivated at 37 °C until OD600 reached the mid-log phase. The bacterial cultures were divided into groups: control, the cultures with antibiotics (1/2 IC_50_ and 2 × IC_50_ concentrations), and the cultures with antibiotics (1/2 IC_50_, 2 × IC_50_ concentrations, and 6 × IC_50_ concentrations) + DPE (128 μg/mL) or BPA (32 μg/mL). After 2 h of cultivation, the bacterial culture was introduced into fresh LB broth at a dilution of one thousandfold. The cell counts (CFU/mL) were then assessed every hour using plate counts until they reached ten times the initial counts. The time taken for the bacterial counts to increase by 1 log in each group can then be calculated.

### 3.9. Membrane Integrity Assay

The enhanced permeability of the outer membrane (OM) was assessed through the measurement of the increasing fluorescence during the uptake kinetics of 1-N-phenyl naphthylamine (NPN) according to Helander and Mattila-Sandholm [43]. Briefly, *E. coli* Kam3-AcrB cells were grown in MH broth medium until the OD600 reached 0.6, and the cell pellet was collected by centrifugation (6000× *g* for 20 min). The cells were then suspended with PBS twice and diluted to a final OD600 of 0.6. The cell suspensions were pre-incubated with cetyltrimethylammonium bromide (CTAB), DPE, DPT, and BPA at RT for 1 h, followed by the addition of NPN (20 µM), and the fluorescence was monitored for 90 min. The NPN fluorescence was measured using a microplate reader with excitation and emission wavelengths set to 375 and 420 nm, respectively.

$$\frac{Fluorescence\;obtained\;from\;tested\;compounds}{Fluorescence\;obtained\;from\;control\;group}=NPN\;Uptake\;Factor\;$$

### 3.10. Cytotoxicity Assay

The cell culture reagents used in this study were acquired from Gibco/Thermo Fisher Scientific Inc. (Bethesda, MD, USA). A sample of 4 × 10^4^ HepG2 cells (obtained from the Bioresource Collection and Research Center (BCRC, Hsinchu, Taiwan; BCRC number: RM60025)) were seeded in a 12-well culture plate and incubated overnight. Cells were exposed to different concentrations of DPE (0, 16, 32, 64, 128, and 256 µg/mL) and BPA (0, 4, 8, 16, 32, and 64 µg/mL) for a duration of 24 h before conducting the cell viability assay. Human hepatic HepG2 cells were maintained and supplemented with 50 units/mL penicillin G and 50 µg/mL streptomycin sulfate as per the instructions provided by Bioresource Collection and Research Center (BCRC, Hsinchu, Taiwan). All the reagents required for cell culture were procured from Gibco/Thermo Fisher Scientific Inc. (Bethesda, MD, USA).

### 3.11. Statistical Analysis

Statistical analysis was performed on the data using SPSS version 12 (Chicago, IL, USA) and the results were displayed as means ± standard deviation. One-way analysis of variance (ANOVA) was employed to assess differences in means among samples, with statistical significance set at *p* < 0.05, and Tukey test was utilized for multiple comparisons of means.

## 4. Conclusions

Only a limited number of EPIs for gram-negative bacteria have been reported to date; additionally, no EPI has been approved for clinical use. In this study, structure-based virtual screening by using a docking program provided an overall assessment of the efflux pump inhibitory activity of the selected 24 compounds against drug-resistant *E. coli*. Our experimental data further showed that DPE and BPA could serve as therapeutic adjuvants, revitalizing the effectiveness of multiple antibiotics against gram-negative pathogens without interrupting cell membrane integrity and exhibiting mild human-cell toxicity. Future studies should aim to clarify how these compounds interact with efflux pumps to inhibit their activity effectively. Additional research is required to validate the therapeutic effectiveness and safety of this active combination in in vivo studies.

## Figures and Tables

**Table 1:** Binding affinity of DPM derivatives against transporter AcrB ^1^.

Docking Parameters	Molecular Docking Results				
	PAβN	DPM	DPE	DPT	BPA
Binding free energy (kcal/mol)	−8.8	−8.1	−8.2	−8.2	−8.5
Number of pseudobond (<4 Å)	42	32	42	43	34
Number of van der Waals contact (<4 Å)	10	8	10	10	8
Number of contact residues ^2^	-	7	8	7	7

^1^ AcrB (PDB: 4DX5-B), ^2^ The number of common contact amino acid residues found in the interface between PAβN and AcrB, PAβN, Phenylalanine-Arginine β-Naphthylamide; DPM, Diphenylmethane; DPE, 2,2-diphenylethanol; DPT, di-*p*-tolyl-methanol; BPA, 4-(benzylphenyl)acetonitrile.

**Table 2:** Protein–ligand interaction residues obtained from the Autodock Vina.

Compound	Interaction Residues ^a^
PAβN	Ser134 ^c^ (2.1 Å), Phe136, Val139, Gln,176 ^c^ (2.1 Å), PHE 178 Phe610, Val612, Phe615 ^b^, Phe617, Phe628 ^b^
DPM	Val139, Phe178 ^b^, Met573, Phe610, Val612, Phe615 ^b^, Phe617, Phe628 ^b^
DPE	Phe136, Val139, Phe178 ^b^, Met573, Phe610, Val612, Phe615 ^b^, Phe617, Ile626, Phe628 ^b^
DPT	Phe136, Val139, Pro326, Met573, Phe610, Val612, Phe615 ^b^, Phe617, Ile626, Phe628 ^b^
BPA	Phe136, Val139, Met573, Phe610, Val612, Phe615 ^b^, Phe617, Phe628

^a^ van der Waals contact distance: <4 Å, pseudobond with AcrB, ^b^ π-π interactions, ^c^ H-bonds.

**Table 3:** Checkerboard assay of DPM derivatives and antibiotics on *E. coli* Kam3-AcrB.

		IC_50_ (μg/mL)		
Compound	Antibiotics	Alone	Combination (Compound/Antibiotic)	MF
DPE	Erythromycin	125	16/31.25	4
	Ciprofloxacin	0.015	128/0.0075	2
	Clarithromycin	87.5	128/21.88	2
	Tetracycline	0.39	128/0.2	2
DPT	Erythromycin	125	4/62.5	2
	Ciprofloxacin	0.015	8/0.0075	2
	Clarithromycin	87.5	4/43.75	2
	Tetracycline	0.39	8/0.2	2
BPA	Erythromycin	125	32/31.25	4
	Ciprofloxacin	0.015	32/0.0075	2
	Clarithromycin	87.5	8/21.88	2
	Tetracycline	0.39	2/0.2	2
DPM	Erythromycin	125	7.81/62.5	2
	Ciprofloxacin	0.015	250/0.0075	2
	Clarithromycin	87.5	250/43.75	2
	Tetracycline	0.39	500/0.098	2
PAβN	Erythromycin	125	10/3.91	32
	Ciprofloxacin	0.015	2.5/0.0038	4
	Clarithromycin	87.5	1.25/10.94	8
	Tetracycline	0.39	0.63/0.049	8

IC_50_, half maximal inhibitory concentration; MF, modulation factor; IC_50_ of DPM (500 µg/mL); DPE (256 µg/mL); DPT (32 µg/mL); BPA (64 µg/mL); PAβN (20 µg/mL).

**Table 4:** EB accumulation of DPM derivatives in *E. coli* Kam3-AcrB.

Compound	Concentration	RFF ± SD
Control	none	0.54 ± 0.06 ^d^
PAβN	20 µg/mL	1.26 ± 0.03 ^c^
DPE	128 µg/mL	2.47 ± 1.02 ^ab^
	64 µg/mL	1.32 ± 0.14 ^c^
	32 µg/mL	1.11 ± 0.00 ^c^
DPT	32 µg/mL	0.49 ± 0.01 ^d^
BPA	64 µg/mL	3.47 ± 0.27 ^a^
	32 µg/mL	1.97 ± 0.23 ^bc^
	16 µg/mL	1.19 ± 0.03 ^c^

RFF, relative final fluorescence; ^a–d^ Different superscript letters indicate statistically significant differences (*p* < 0.05)

**Table 5:** The post-antibiotic effect of DPE and BPA with erythromycin.

Regimen	Mean PAE (h) ± SD		
	1/2 IC50	2 IC50	6 IC50
Erythromycin	0.61 ± 0.01	1.00 ± 0.33	1.10 ± 0.38
Erythromycin + DPE	1.09 ± 0.32	1.13 ± 0.35	1.44 ± 0.2
Erythromycin + BPA	1.08 ± 0.37	1.21 ± 0.58	1.39 ± 0.58

Data are expressed as mean ± SD (*n* = 3).

## Data Availability

The data presented in this study are available on request from the corresponding author due to privacy.

## Supplementary Materials

The following are available online at https://www.mdpi.com/article/10.3390/antibiotics13070628/s1, Figure S1. Experimental design schematic diagram for the investigation of diphenylmethane-scaffold seaweed compounds as potential efflux pump inhibitors against drug-resistant *Escherichia coli*. Figure S2. EB accumulation of DPM derivatives in E. coli Kam3 AcrB. (**A**) DPE, (**B**) DPT, (**C**) BPA. Data are expressed as mean ± SD (*n* = 3).
