# Supplementary material for: Screening and Evaluation of Potential Efflux Pump Inhibitors with a Seaweed Compound Diphenylmethane-Scaffold against Drug-Resistant Escherichia coli
Source: Antibiotics (Basel). 2024 Jul 5;13(7):628. doi: 10.3390/antibiotics13070628 (PMC11274134; doi:10.3390/antibiotics13070628)
Supplement: Supplementary file 1 [file antibiotics-13-00628-s001.zip › antibiotics-3061775-supplementary.pdf]

antibiotics

MDPI

Supplementary Materials

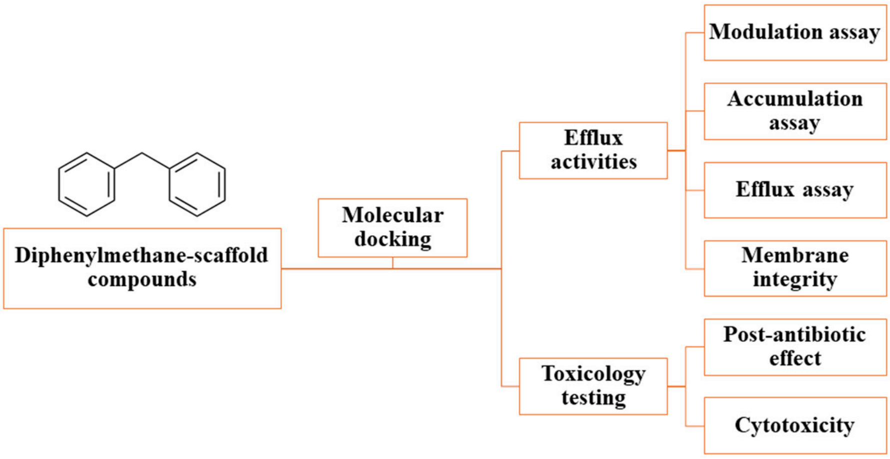

Figure S1. Experimental design schematic diagram for the investigation of diphenylmethane-scaffold seaweed compounds as potential efflux pump inhibitors against drug-resistant *Escherichia coli*.

(A)

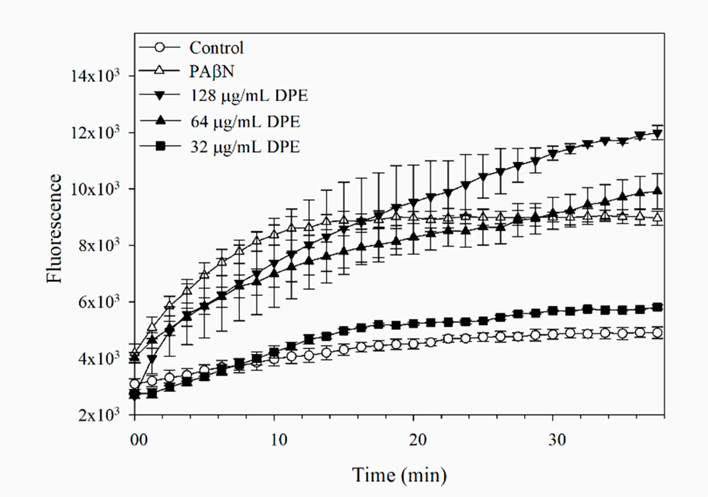

(B)

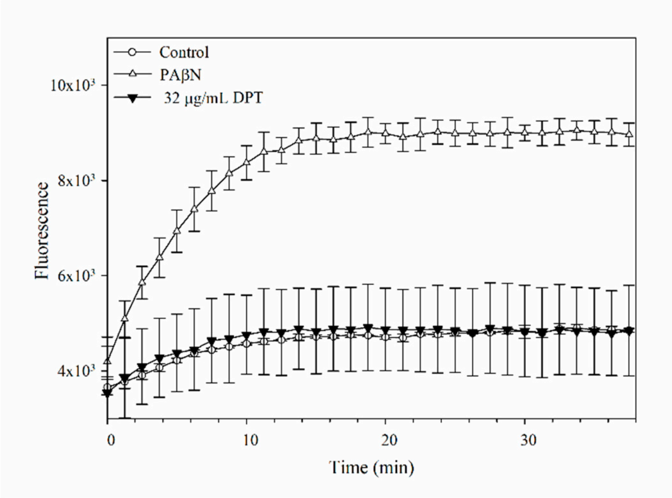

(C)

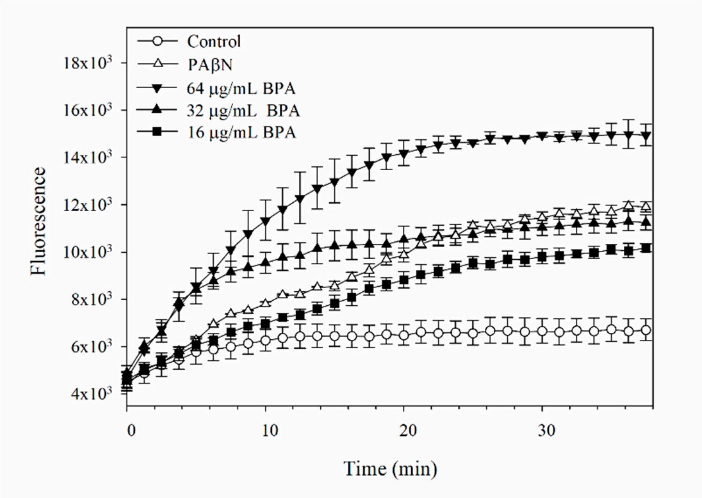

Figure S2. EB accumulation in the presence of (**A**) DPE, (**B**) DPT, and (**C**) BPA in *E. coli* Kam3 AcrB. Data are expressed as mean ± SD (*n* = 3).
